# Haemozoin Induces Early Cytokine-Mediated Lysozyme Release from Human Monocytes through p38 MAPK- and NF-kappaB- Dependent Mechanisms

**DOI:** 10.1371/journal.pone.0039497

**Published:** 2012-06-18

**Authors:** Manuela Polimeni, Elena Valente, Elisabetta Aldieri, Amina Khadjavi, Giuliana Giribaldi, Mauro Prato

**Affiliations:** Dipartimento di Genetica, Biologia e Biochimica, Facoltà di Medicina e Chirurgia, Università degli Studi di Torino, Torino, Italy; Jr, Johns Hopkins Bloomberg School of Public Health, United States of America

## Abstract

Malarial pigment (natural haemozoin, HZ) is a ferriprotoporphyrin IX crystal produced by *Plasmodium* parasites after haemoglobin catabolism. HZ-fed human monocytes are functionally compromised, releasing increased amounts of pro-inflammatory molecules, including cytokines, chemokines and cytokine-related proteolytic enzyme Matrix Metalloproteinase-9 (MMP-9), whose role in complicated malaria has been recently suggested. In a previous work HZ was shown to induce through TNFalpha production the release of monocytic lysozyme, an enzyme stored in gelatinase granules with MMP-9. Here, the underlying mechanisms were investigated. Results showed that HZ lipid moiety promoted early but not late lysozyme release. HZ-dependent lysozyme induction was abrogated by anti-TNFalpha/IL-1beta/MIP-1alpha blocking antibodies and mimicked by recombinant cytokines. Moreover, HZ early activated either p38 MAPK or NF-kappaB pathways by inducing: p38 MAPK phosphorylation; cytosolic I-kappaBalpha phosphorylation and degradation; NF-kappaB nuclear translocation and DNA-binding. Inhibition of both routes through selected molecules (SB203580, quercetin, artemisinin, parthenolide) prevented HZ-dependent lysozyme release. These data suggest that HZ-triggered overproduction of TNFalpha, IL-1beta and MIP-1alpha mediates induction of lysozyme release from human monocytes through activation of p38 MAPK and NF-kappaB pathways, providing new evidence on mechanisms underlying the HZ-enhanced monocyte degranulation in *falciparum* malaria and the potential role for lysozyme as a new affordable marker in severe malaria.

## Introduction

Malaria is one of the most common parasitic diseases in the world and causes over 1 million deaths per year [1], [2]. The causative agent (*Plasmodium*) has a biological cycle in the mosquito vector and in the human host; the most virulent among the human malaria parasites is *Plasmodium falciparum*, which is responsible for the bulk of the malaria-related morbidity and mortality [2], [3]. Symptoms of uncomplicated malaria include fever, headache, and vomiting. The major complications of severe malaria include cerebral malaria, pulmonary edema, acute renal failure or severe anaemia [4]–[7]. It mainly affects children under 5 years of age and pregnant women, and can be fatal [8]. It is characterized by the binding of infected red blood cell (IRBC) to the vascular endothelium (cytoadherence) and to non-infected erythrocytes (rosetting). The accumulation of IRBCs and non-infected RBCs generates a reduction in the blood flow of the microvasculature, which results in tissue hypoxia and necrosis [9]. There is growing evidence that the combination of both parasite and host factors is involved in the pathogenesis of severe malaria. Among parasite products, natural haemozoin (HZ, malaria pigment) may be a candidate. HZ is a lipid-bound ferriprotoporphyrin IX crystal produced by late stages (trophozoites and schizonts) of *Plasmodium* parasites, and is avidly phagocytosed by human phagocytic cells such as monocytes and macrophages [10]. As a result of phagocytosis, several functions of monocytes are seriously compromised, including repeated phagocytosis [11], bacterial killing abilities [12], oxidative burst [11], MHC Class II expression and antigen presentation [13], maturation to dendritic cells [14], and coordination of erythropoiesis [15]. However, impaired HZ-laden human monocytes do not undergo apoptosis: apparently, HZ-dependent enhanced expression of anti-apoptotic HSP-27 leads to prolonged monocyte survival, thereby contributing to maintain the impaired monocytes in the bloodstream [16], [17]. Moreover, HZ-fed monocytes show enhanced gene expression of a large number of pro-inflammatory molecules, including cytokines (TNFalpha, IL-1beta, IL-1RA) and chemokines (MIP-1alpha and beta, GROalpha, beta and gamma, MCP-1, IL-8, ENA-78) [16]. HZ also upregulates expression and activity of few monocytic enzymes. As more extensively reviewed by Prato and Giribaldi [18], HZ was shown in a series of recent works from our group to increase RNA/protein expression, protein release and proteolytic activity of matrix metalloproteinase-9 (MMP-9), a cytokine-related proteolytic enzyme. Additionally, in a recent study we showed that 2 h after phagocytosis, HZ promoted production and release of high amounts of lysozyme [19], an antibacterial protein defined by its ability to hydrolyse beta-1,4-glycosidic linkage between N-acetylmuramic acid and N-acetylglucosamine of peptidoglycan in the cell wall of bacteria (muramidase activity) [20]. This finding appears particularly relevant in the context of research for early diagnosis markers of severe malaria, since plasma lysozyme levels in malaria patients have been related to disease severity [21], [22]; additionally, it should be coupled with the ground-breaking evidence recently published by Kajla et al. showing that in *Anopheles* vectors the mosquito lysozyme homologue facilitates the development of *Plasmodium berghei* and *falciparum* through direct binding to parasite oocysts [23]. In the present work, the mechanisms underlying HZ-dependent human lysozyme enhancement in monocytes were investigated, focusing on the dependence on production of pro-inflammatory molecules and activation of p38 mitogen-activated protein kinase (MAPK) and nuclear factor-kappaB (NF-kappaB) pathways.

## Materials and Methods

### Materials

Unless otherwise stated, reagents were obtained from Sigma-Aldrich, St. Louis, MO. Sterile plastics were from Costar, Cambridge, UK; Cell culture media RPMI, TRIzol, M-MLV, oligo-dT, sense and anti-sense primers, Platinum Taq DNA Polymerase were from Invitrogen, Carlsbad, CA; Panserin 601 monocyte medium was from PAN Biotech, Aidenbach, Germany; Percoll was from Pharmacia, Uppsala, Sweden; Diff-Quik parasite stain was from Baxter Dade AG, Dudingen, Switzerland; enzyme-linked immunoadsorbent assay (ELISA) kits for TNFalpha and IL-1beta assay were from Cayman, Ann Arbor, MI; blocking anti-hTNFalpha/IL-1beta antibodies and rhTNFalpha/IL-1beta were from Merck, Darmstadt, Germany; ELISA kits for MIP-1alpha, anti-hMIP-1alpha blocking antibodies and rhMIP-1alpha were from R&D Systems, Minneapolis, MN; p38 MAPK inhibitor SB203580 was from Cell Signaling Technology, Danvers, MA; bicinchoninic acid protein assay was from Pierce, Rockford, IL; anti-I-kappaBalpha and anti-NF-kappaB-p65 polyclonal antibodies and anti-P-p38 MAPK, anti-p38 MAPK, anti-P-I-kappaBalpha and anti-NF-kappaB-p50 monoclonal antibodies were from Santa Cruz Biotechnology, Santa Cruz, CA; electrophoresis reagents were from Bio-Rad Laboratories, Hercules, CA; DNA-free kit was from Ambion, Austin, TX; Beacon Designer 7.0 software was from Premier Biosoft International, Palo Alto, CA; dNTPs were from Applied Biosystem, Foster City, CA.

### Culturing of P. Falciparum and Isolation of HZ

*P. falciparum* parasites (Palo Alto strain, mycoplasma-free) were kept in culture as described [24], [25]. To isolate HZ, IRBCs (4–8% parasitemia) were washed twice with serum-free culture medium, re-suspended to 25% haematocrit and fractionated on a discontinuous Percoll/6% mannitol (wt/vol) gradient (0, 40, 80%); after centrifugation at 1075*g*, HZ was collected at the top of the 0–40% gradient interphase, extensively washed with (hypotonic) 10 mM phosphate buffer (pH 8.0) containing 10 mM mannitol and stored at −20°C; repeated washings with 10 mM hypotonic PBS of the isolated HZ was done to lyse eventual residual contaminating cells and remove cell remnants [26]. The haem content of a weighed amount of HZ dissolved in 0.1 M NaOH was determined by reading the absorbance at 385 nm (6.1×10^4^ M cm^−1^, Soret band). Beta-haematin (sHZ, synthetic HZ) was prepared in methanol as previously described [24]. For delipidized HZ (dHZ), lipid extraction was performed as previously reported [24].

### Preparation and Handling of Monocytes

Human monocytes were separated by Ficoll centrifugation from freshly collected buffy coats discarded from blood donations by healthy adult donors of both sexes provided by the local blood bank (AVIS, Associazione Volontari Italiani Sangue, Torino, Italy); informed consent from donors was obtained by AVIS, and human specimens were further handled anonymously by our group [27]. Separated lympho/monocytes were re-suspended in RPMI 1640 medium and plated on wells of 6-well plates. Each well received 2 ml of cell suspension containing 8×10^6^ cells/ml in RPMI 1640. The plates were incubated in a humidified CO_2/_air-incubator at 37°C for 60 min. Thereafter non-adherent cells were removed by 3 washes with RPMI 1640 and adherent cells re-incubated at 37°C overnight in RPMI 1640. Shortly before starting phagocytosis, wells were washed with RPMI 1640 and Panserin 601 medium was added (2 ml/well).

### Pre-Selection of NF-KappaB-Quiescent Monocytes by Flow Cytometry and Real Time RT-PCR

Before starting experiments, a pre-selection of cell populations was taken as a precautionary measure, as previously described [27]. Briefly, cell cultures isolated through Ficoll separation were analyzed by flow cytometry. Only cell populations showing at least 70% monocytes were used for following experiments. Additionally, in order to avoid the use of NF-kappaB pre-activated monocytes, cells were analyzed by Real Time RT-PCR: in each cell preparation a cell aliquot was stimulated or not with LPS (1 µg/ml) for 4 h, and TNFalpha RNA production was measured in lysates by Real Time RT-PCR. GAPDH was used as house-keeping gene. Only unstimulated monocyte populations (“NF-kappaB-quiescent" cells) showing at least a 3-PCR-cycles gap of cDNA amplification between controls and LPS-stimulated cells were used for subsequent experiments.

### Phagocytosis of Opsonized Latex Particles or HZ and Treatment with Recombinant Cytokines, Blocking Antibodies and Chemical Inhibitors

Latex particles and HZ washed once and finely dispersed at 30% (v/v) in PBS were added to the same volume of fresh human AB serum (AVIS blood bank) and incubated for 30 min at 37°C to reach opsonization as described [27]. Phagocytosis was started by adding to adherent monocytes opsonized latex particles, HZ, sHZ or dHZ (50 RBC equivalents, in terms of haem content, per monocyte). The plates were then incubated in Panserin 601 medium in a humidified CO_2_/air-incubator at 37°C for 2 h. After the end of the phagocytic period, cells were checked by optical microscopy: as an average, HZ-containing monocytes were 25–35% among the total cells, a percentage compatible with *in vivo* levels measured in patients with severe malaria showing high parasitaemia [28]. Therefore, monocytes were washed and incubated in Panserin 601 medium in a humidified CO_2_/air-incubator at 37°C for the indicated times in the presence/absence of: anti-hTNFalpha, anti-hIL-1beta, or anti-hMIP-1alpha blocking antibodies (all 30 ng/ml); rhTNFalpha, rhIL-1beta, or rhMIP-1alpha (all 20 ng/ml); p38 MAPK inhibitor SB203580, quercetin, artemisinin or parthenolide (all 10 microM except quercetin: 15 microM).

### Assay of Lysozyme Activity

Lysozyme released from adherent monocytes was assayed as previously described [19]. Briefly, monocyte supernatants were incubated with suspensions of *Mycrococcus Lysodeikticus* at an OD_450nm_ of 1 in 0.4 M phosphate buffered saline, pH 6.7, at 37°C. The change in OD_450nm_ was measured after 30 min incubation. A standard calibration curve was generated with purified chicken egg lysozyme. One enzyme unit of enzyme activity corresponded to a decrease of 0.001 OD units each minute.

### Assay of TNFalpha, IL-1beta, and MIP-1alpha Production

The levels of soluble TNFalpha, IL-1beta, and MIP-1alpha were assayed in monocyte supernatants by specific ELISA. A standard calibration curve was generated with rhTNF, rhIL-1beta, and rhMIP-1alpha, according to the manufacturer’s instructions.

### Isolation of Cytosolic and Nuclear Protein Fractions in Cell Lysates

Cells were mechanically scraped in PBS and washed, then resuspended in lysis buffer (15 mM KCl, 10 mM HEPES, 2 mM MgCl_2_, 0.1 mM EDTA, 1 mM PMSF, 1 mM DTT, 10 µg/ml aprotinin, 2 µg/ml leupeptin, 0.1% NP-40, pH 7.6). Cell suspensions were then incubated for 15 min on ice with occasional vortexing, and centrifuged for 30 s to pellet nuclei. Supernatants with cytosolic proteins were collected for following experiments. Nuclei were rinsed with wash buffer (2 mM KCl, 25 mM HEPES, 0.1 mM EDTA, 1 mM PMSF, 1 mM DTT, 10 µg/ml aprotinin, 2 µg/ml leupeptin, pH 7.6) and incubated at 4°C for 20 min. Nuclear extracts were then prepared by centrifugation at 20,000*g* for 15 min in lysis buffer (25 mM HEPES, 0.1 mM EDTA, 20% glycerol, pH 7.6) and used for subsequent experiments. Protein concentration was determined using a bicinchoninic acid assay.

### Assay of p38 MAPK, P-p38 MAPK, I-KappaBalpha, P-I-KappaBalpha, p50(NF-KappaB) and p65(NF-KappaB) Protein Levels by Western Blotting (WB)

Nuclear or cytosolic proteins were separated on 10 or 12% polyacrylamide gel, blotted on polyvinylidene difluoride membrane, and probed with different primary antibodies: polyclonal anti-I-kappaBalpha, anti-NF-kappaB-p65 and anti-actin antibodies, or monoclonal anti-P-p38 MAPK, anti-p38 MAPK, anti-P-I-kappaBalpha and anti-NF-kappaB-p50 antibodies. After staining with secondary anti-rabbit or anti-mouse horse-radish peroxidase-conjugated antibodies, bands were visualized by ECL staining. Every blot was re-used for multiple times by stripping and re-staining. Firstly, all blots were analyzed for actin protein levels (housekeeping gene, data not shown), in order to verify that equal protein amounts were present in each lane. Therefore, all blots were stripped, and grouped in three separate batches. Every batch was used twice, to analyze two different proteins: the first batch was used to study both p38 MAPK and P-p38 MAPK proteins; the second one was used for P-I-kappaBalpha and I-kappaBalpha proteins; and the third one for NF-kappaB-p65 and NF-kappaB-p50 proteins.

### Assay of NF-KappaB Complex Nuclear Translocation by Electrophoretic Mobility Shift Assay (EMSA)

Probes containing the NF-kappaB oligonucleotide consensus sequence were labelled with ^32^P (3,000 Ci/mmol, 250 µCi) using T4 polinucleotide kinase. Nuclear extracts were incubated for 20 min with 20,000 cpm of ^32^P-labeled double-stranded oligonucleotide at 4°C in a reaction mixture containing 10 µg/ml BSA, 10x buffer (100 mM KCl, 20 mM HEPES, 0.5 mM EDTA, 2 mM DTT, 0.1 PMSF, 20% glycerol, 0.25% NP-40, pH 7.6), 5x buffer (300 mM KCl, 100 mM HEPES, 10 mM DTT, 100 µM PMSF, 20% Ficoll, pH 7.6) and 1 µg/ml poly(dI-dC). The DNA-protein complex was separated on a non denaturing 4% polyacrylamide gel. After electrophoresis, the gel was dried and autoradiographed by overnight exposure to X-ray film.

### Statistical Analysis

For each set of experiments, data are shown as means + SEM (lysozyme assay and ELISA) or as a representative image (WB and EMSA) of three independent experiments with similar results. All data were analyzed by a one-way Analysis of Variance (ANOVA) followed by Tukey’s post-hoc test (software: SPSS 16.0 for Windows, SPSS Inc., Chicago, IL).

## Results

### The Lipid Moiety of HZ Promotes Early Time-Dependent Release of Lysozyme from Human Adherent Monocytes

**Figure 1:**
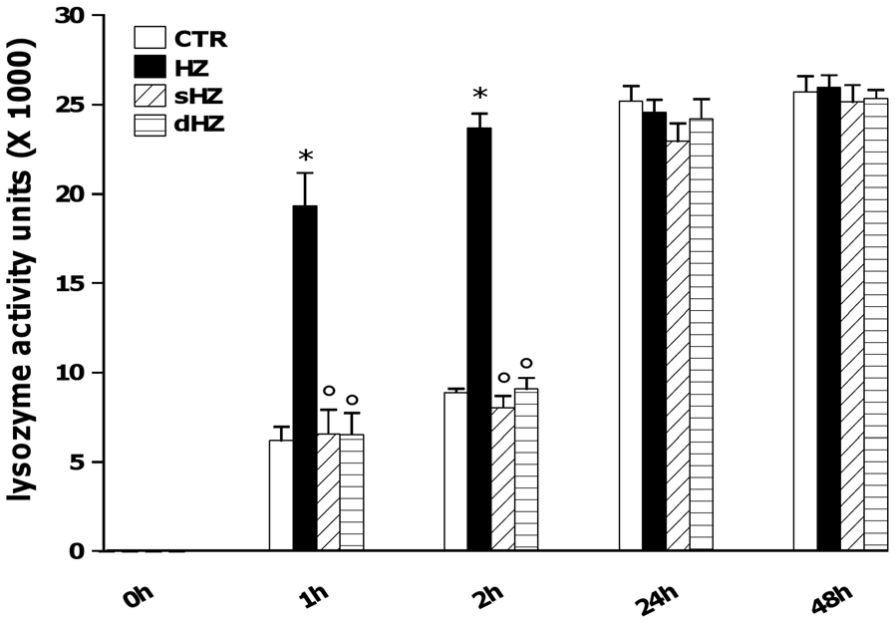
Early time-dependent induction of lysozyme release from HZ-fed human adherent monocytes: role of the lipid moiety of HZ. Cells were unfed (control cells, CTR) and fed with HZ, sHZ or dHZ for 2 h; then, lysozyme levels in cell supernatants were monitored 0, 1, 2, 24 and 48 h after the end of phagocytosis by spectrometric assay. Data are means + SEM of three independent experiments. Lysozyme release from monocytes is indicated as enzyme activity units measured in a 2 ml-well of cell supernatants. All data were evaluated for significance by ANOVA. Vs CTR *p<0.0001; Vs HZ °p<0.0001.

In a previous work, we showed that HZ induced lysozyme release from human monocytes 2 h after the end of phagocytosis [19]. In order to clarify whether a time-dependence of this effect occurred, and to identify what component of HZ among ferric and lipid moieties was responsible, human adherent monocytes were unfed (control cells, CTR) or fed with HZ, sHZ and dHZ for 2 h. After termination of phagocytosis cells were washed and incubated for 48 additional h. Cell supernatants were collected 0, 1, 2, 24 and 48 h after the end of phagocytosis, and lysozyme activity levels were measured by a specific spectrometric assay. Results are shown in Figure 1. CTR cells released basal levels of lysozyme (≅6000 and ≅9000 activity units at 1^st^ and 2^nd^ h, respectively, and ≅25000 activity units at both 24^th^ and 48^th^ h). After phagocytosis of HZ, lysozyme levels in cell supernatants were almost double than those released from CTR cells after 1 and 2 h (p<0.0001); on the contrary, no significant differences between CTR and HZ were observed at longer incubation times. Moreover, phagocytosis of lipid-free HZ (sHZ and dHZ) did not reproduce HZ effects, and lysozyme levels in cell supernatants were similar to those of unfed cells at all times of the observational period.

### Role of Pro-Inflammatory Molecules TNFalpha, IL-1beta, and MIP-1alpha in HZ-Dependent Early Induction of Lysozyme Release from Human Adherent Monocytes

**Figure 2:**
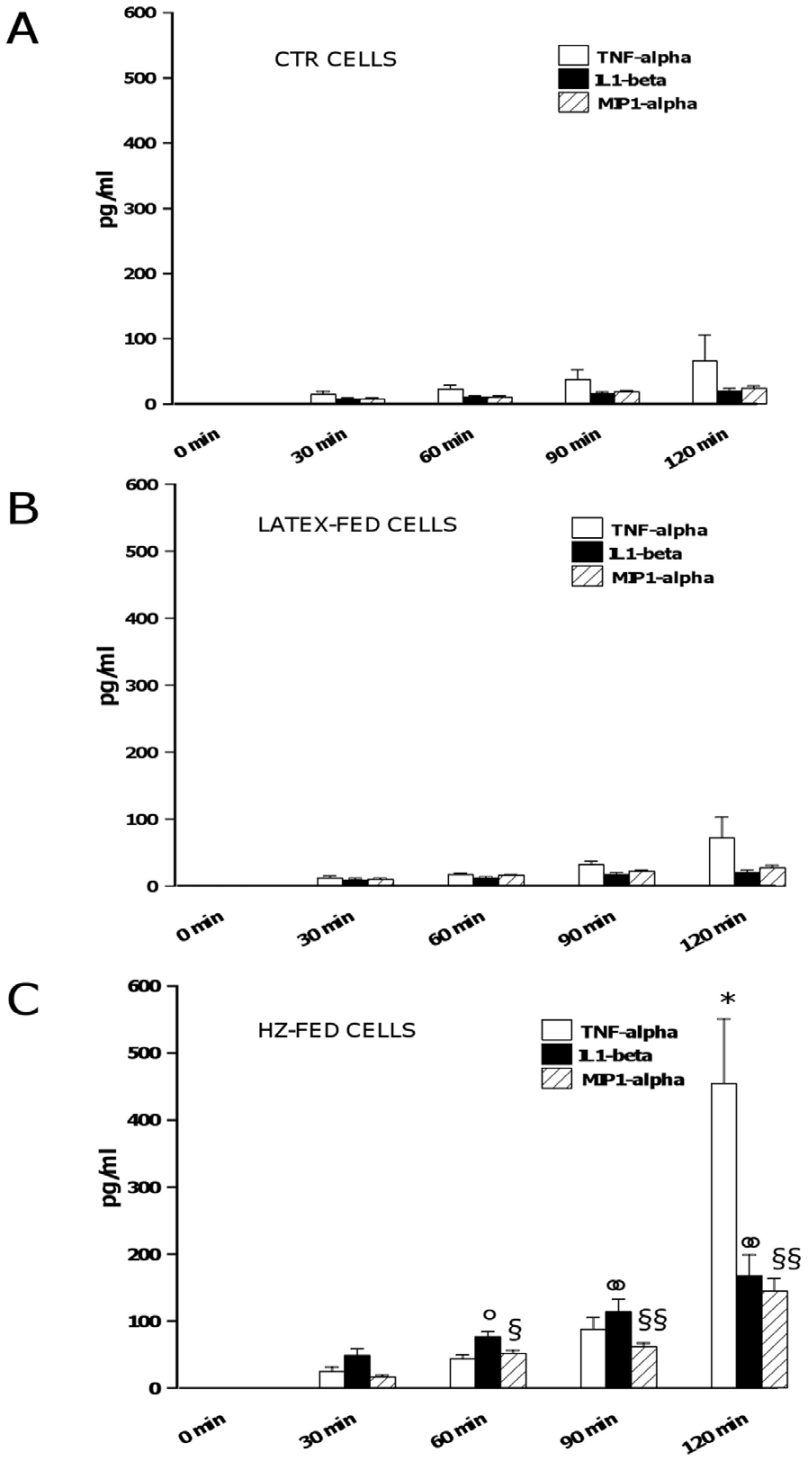
Early time-dependent enhanced production of TNFalpha, IL-1beta, and MIP-1alpha by HZ-fed human adherent monocytes. Cells were unfed (CTR cells) (Panel A), fed with latex particles (latex-fed cells) (Panel B), or fed with HZ (HZ-fed cells) (Panel C) for 2 h; afterwards, production of TNFalpha, IL-1beta, and MIP-1alpha was monitored every 30 min in cell supernatants up to 2 h. Data are means + SEM of three independent experiments. Production of pro-inflammatory molecules is indicated as pg/ml. All data were evaluated for significance by ANOVA. HZ-fed vs CTR cells: *p<0.0001 for TNFalpha (120 min); °p<0.01 for IL-1beta (60 min), °°p<0.0001 for IL-1beta (90 and 120 min); §p<0.001 for MIP-1alpha (60 min), §§p<0.0001 for MIP-1alpha (90 and 120 min).

**Figure 3:**
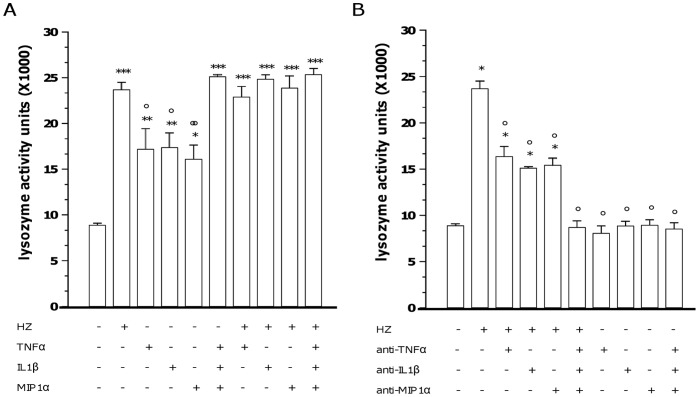
Role of TNFalpha, IL-1beta and MIP-1alpha in HZ-dependent induction of lysozyme release from human monocytes. Cells were unfed (negative controls) or fed with HZ (positive controls) for 2 h. Afterwards, cells were incubated for 2 h alone and with a single dose (20 ng/ml) or a combination of rhTNFalpha, rhIL-1beta and rhMIP-1alpha (Panel A, mimicking approach); alternatively, cells were incubated for 2 h alone and with a single dose (30 ng/ml) or a combination of anti-hTNFalpha, anti-hIL-1beta or anti-hMIP-1alpha blocking antibodies (Panel B, blocking approach). Thereafter, lysozyme release was measured by spectrometric assay. Data are means + SEM of three independent experiments. Lysozyme release from monocytes is indicated as enzyme activity units measured in a 2 ml-well of cell supernatants. All data were evaluated for significance by ANOVA. Panel A: Vs unstimulated cells (column 1) *p<0.05, **p<0.01, ***p<0.0001; Vs untreated HZ-fed cells (column 2) °p<0.05, °°p<0.01. Panel B: Vs unstimulated cells (column 1) *p<0.0001; Vs untreated HZ-fed cells (column 2) °p<0.0001.

HZ-induced lysozyme release from human monocytes was previously shown to be partially dependent on HZ-enhanced TNFalpha production [19]. Thus, the possible role for two additional HZ-enhanced pro-inflammatory molecules (IL-1beta and MIP-1alpha) as soluble mediators of early lysozyme release was investigated, in order to verify whether they could synergise with TNFalpha. Human adherent monocytes were unfed (CTR cells), fed with latex particles (control meals, latex-fed cells), or fed with HZ (HZ-fed cells) for 2 h. After termination of phagocytosis cells were washed and incubated for 2 additional h. Cell supernatants were collected every 30 min up to 2 h after the end of phagocytosis, and TNFalpha, IL-1beta, and MIP-1alpha production was measured by ELISA, as a preliminary assessment of protein levels. Results are shown in Figure 2. CTR (Panel A) and latex-fed (Panel B) cells released time-dependently low levels of all the pro-inflammatory molecules studied, with less than 100 pg/ml for TNFalpha and less than 30 pg/ml for either IL-1beta or MIP-1alpha at the latter time-point evaluated in both conditions. After phagocytosis of HZ (Panel C, HZ-fed cells), the time-dependent production of IL-1beta was significantly higher than CTR/latex-fed cells, reaching up to 150 pg/ml at the latter time-point evaluated (p<0.0001). TNFalpha and MIP-1alpha production in HZ-fed cells was not significantly different than CTR/latex-fed cells at the earlier time-points measured, whereas it became significantly higher during the observational period, reaching up to 500 pg/ml for TNFalpha (p<0.0001) and up to 150 pg/ml for MIP-1alpha (p<0.0001) at the latter time-point evaluated. Further experiments were performed to investigate and compare the role of pro-inflammatory molecules IL-1beta, MIP-1alpha, and TNFalpha (alone or combined) in the HZ-dependent early induction of lysozyme release from monocytes. Two different approaches were adopted: in the first one, CTR/HZ-fed cells were incubated for 2 h either with a single dose (20 ng/ml) of rhTNFalpha, rhIL-1beta and rhMIP-1alpha or with a combination of them (mimicking approach); alternatively, cells were incubated for 2 h either with a single dose (30 ng/ml) of anti-hTNFalpha, anti-hIL-1beta or anti-hMIP-1alpha blocking antibodies or with a combination of them (blocking approach). Thereafter, the levels of lysozyme activity were measured in monocyte supernatants by spectrometric assay. Results are shown in Figure 3 (Panel A: mimicking approach; Panel B: blocking approach). The levels of lysozyme released from unfed cells and HZ-fed cells should be referred as negative and positive controls, respectively; additionally, the values obtained by using rhTNFalpha or anti-TNFalpha antibodies (already published previously in [19]) serve as internal controls between single and combined treatments. As shown in Panel A, the effect of HZ was partially mimicked by the addition of single doses of rhTNFalpha (p<0.01), rhIL-1beta (p<0.01), and rhMIP-1alpha (p<0.05) to unfed cells, whereas a totally HZ-mimicking effect was reached by adding a full combination of all recombinant cytokines (p<0.0001). None of treatments with recombinant cytokines did affect basal lysozyme release from HZ-fed cells (p not significant). As shown in Panel B, the effect of HZ on lysozyme release was reduced by the addition of single doses of anti-hTNFalpha (p<0.0001), anti-hIL-1beta (p<0.0001), and anti-hMIP-1alpha (p<0.0001) blocking antibodies and totally abrogated by adding a full combination of all blocking antibodies to HZ-fed cells (p<0.0001). None of treatments with blocking antibodies did affect basal lysozyme release from unfed cells (p not significant).

### Involvement of p38 MAPK Pathway in HZ-Dependent Early Induction of Lysozyme Release from Human Adherent Monocytes

**Figure 4:**
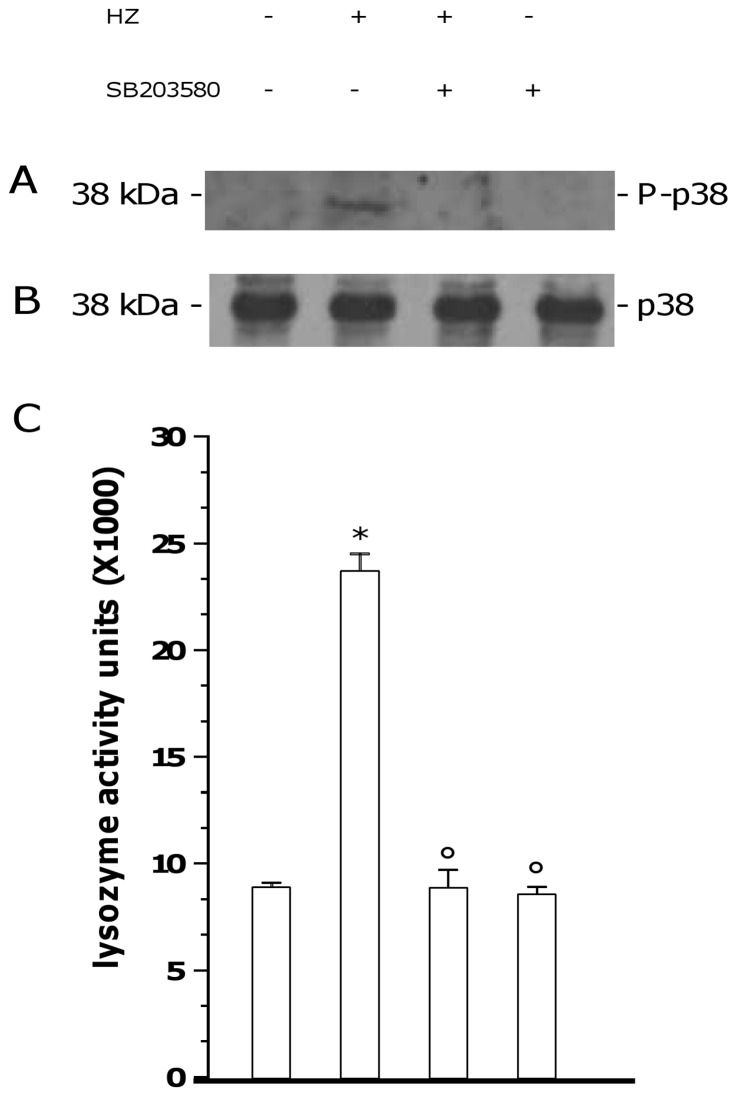
Involvement of p38 MAPK pathway in HZ-dependent induction of lysozyme release from human adherent monocytes. Cells were unfed (negative controls) or fed with HZ (positive controls) for 2 h; after phagocytosis, cells were incubated for 2 h alone and with 10 microM p38 MAPK synthetic inhibitor SB203580. Therefore, p38 MAPK protein expression and phosphorylation was evaluated by WB in cell lysates (Panels A and B), whereas lysozyme release was measured by spectrometric assay in cell supernatants (Panel C). Results are shown as a representative blot (A–B) or means + SEM (C) of three independent experiments. Lysozyme release from monocytes is indicated as enzyme activity units measured in a 2 ml-well of cell supernatants. In lysozyme release studies, data were also evaluated for significance by ANOVA. Vs unstimulated cells (column 1) *p<0.0001; Vs untreated HZ-fed cells (column 2) °p<0.0001.

Human adherent monocytes were unfed or fed with HZ for 2 h and then incubated for 2 additional h in the presence/absence of p38 MAPK inhibitor SB203580 (10 microM). Therefore, p38 MAPK protein expression and phosphorylation were evaluated by WB in cell lysates, whereas lysozyme release was measured by spectrometric assay in cell supernatants. Results are shown in Figure 4 (panel A: p38 MAPK phosphorylation; panel B: p38 MAPK expression; Panel C: lysozyme release). None of treatments did affect the basal levels of p38 MAPK. Phosphorylation of p38 MAPK protein was not observed in unstimulated monocytes, whereas it was induced after phagocytosis of HZ; as expected, SB203580 prevented HZ-dependent p38 MAPK phosphorylation, without affecting unfed cells. Moreover, the HZ-dependent early induction of lysozyme release (p<0.0001) was abrogated by p38 MAPK inhibitor (p<0.0001), which did not affect basal lysozyme levels of unfed cells (p not significant).

### Involvement of NF-KappaB Pathway in HZ-Dependent Early Induction of Lysozyme Release from Human Adherent Monocytes

**Figure 5:**
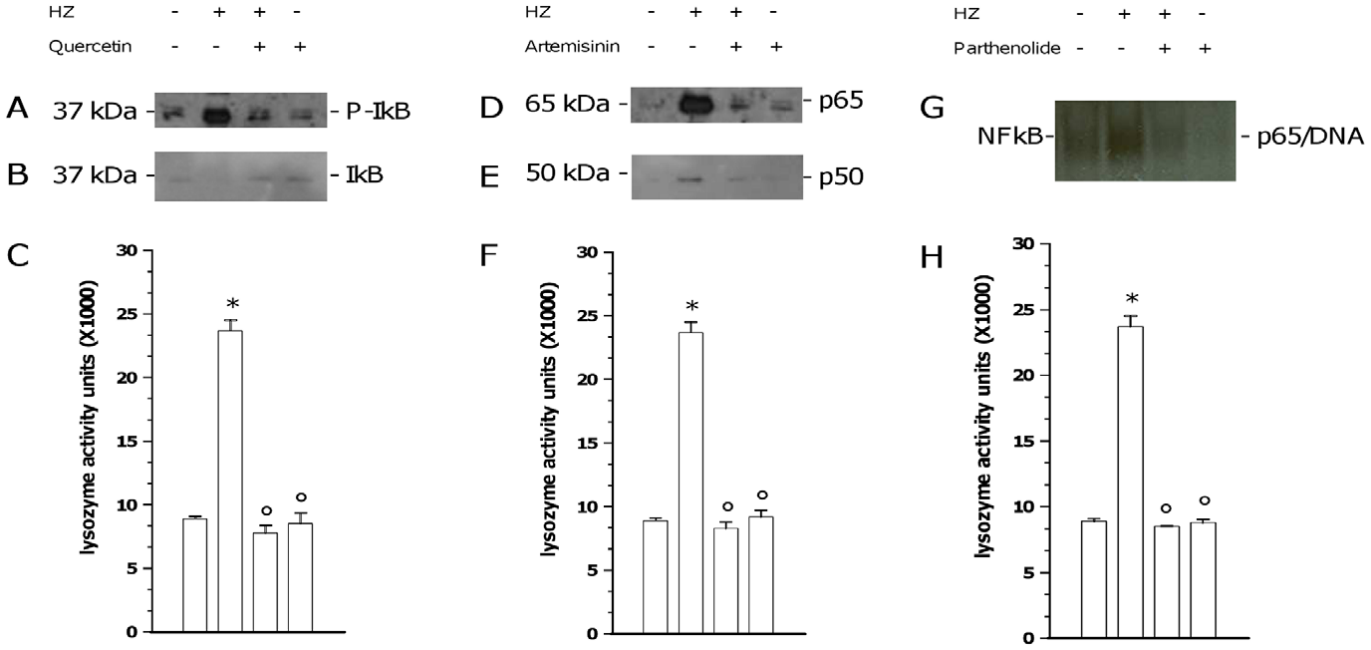
Involvement of NF-kappaB pathway in HZ-dependent induction of lysozyme release from human adherent monocytes. Cells were unfed (negative controls) or fed with HZ (positive controls) for 2 h; after phagocytosis, cells were incubated for 2 h alone and with: 15 microM quercetin, an inhibitor of cytosolic I-kappaBalpha protein phosphorylation (Panels A–C); 10 microM artemisinin, an inhibitor of p65 and p50 NF-kappaB subunits nuclear translocation (Panels D–F); and 10 microM parthenolide, an inhibitor of DNA/NF-kappaB complex binding (Panels G–H). Therefore, I-kappaBalpha protein phosphorylation (A) and degradation (B), along with p65 (D) and p50 (E) nuclear translocation were evaluated by WB in cytosolic and nuclear fractions of cell lysates, respectively; DNA/NF-kappaB complex binding (G) was evaluated by EMSA in nuclear fraction of cell lysates; and lysozyme release was measured by spectrometric assay in cell supernatants (C, F, H). Results are shown as representative images (WB and EMSA studies) or means + SEM (lysozyme release studies) of three independent experiments. Lysozyme release from monocytes is indicated as enzyme activity units measured in a 2 ml-well of cell supernatants. In lysozyme release studies, data were also evaluated for significance by ANOVA. Panel C: Vs unstimulated cells (column 1) *p<0.0001; Vs untreated HZ-fed cells (column 2) °p<0.0001. Panel F: Vs unstimulated cells (column 1) *p<0.0001; Vs untreated HZ-fed cells (column 2) °p<0.0001. Panel H: Vs unstimulated cells (column 1) *p<0.0001; Vs untreated HZ-fed cells (column 2) °p<0.0001.

Human adherent monocytes were unfed or fed with HZ for 2 h and then incubated for 2 additional h in the presence/absence of three molecules which have been reported to block NF-kappaB signaling at different levels: 15 microM quercetin, inhibitor of I-kappaBalpha phosphorylation and subsequent degradation [29]; 10 microM artemisinin, inhibitor of NF-kappaB nuclear translocation [30]; and 10 microM parthenolide, inhibitor of NF-kappaB binding to DNA [31]. Therefore, cell supernatants were collected and cytosolic and nuclear fractions were isolated from cell lysates. Cytosolic I-kappaBalpha protein phosphorylation and degradation, along with nuclear translocation of p65 and p50 NF-kappaB subunits were evaluated by WB; nuclear DNA/NF-kappaB complex binding was evaluated by EMSA; lysozyme release into supernatants was measured by spectrometric assay. Results are shown in Figure 5. Phosphorylation (Panel A) and degradation (Panel B) of I-kappaBalpha protein were not observed in unstimulated monocytes, while they were suddenly induced after phagocytosis of HZ. Quercetin prevented HZ-dependent I-kappaBalpha phosphorylation and degradation, without affecting unfed cells. P65 (Panel D) and p50 (Panel E), two NF-kappaB subunits, were not found in the nuclear fraction of unstimulated cell lysates, while they showed up after phagocytosis of HZ. Artemisinin prevented HZ-dependent p65 and p50 NF-kappaB nuclear translocation, without affecting unfed cells. Binding of NF-kappaB complex to DNA (Panel G) was absent in unstimulated monocytes, while it was promoted by phagocytosis of HZ. Parthenolide prevented HZ-dependent NF-kappaB/DNA binding, without affecting unfed cells. The HZ-dependent early induction of lysozyme release (p<0.0001) was abrogated by all NF-kappaB inhibitors (p<0.0001), which did not affect basal lysozyme levels of unfed cells (Panels C, F, H; p not significant).

## Discussion

Three major distinct lysozyme types showing high level of homology have been identified in the animal kingdom: c-type (chicken-type), present in several members of the Chordata, including humans, and different classes of the Arthropoda, including mosquitoes; g-type (goose-type), in few members of the Chordata and in some bivalve mollusks; and i-type (invertebrate-tipe), in the Invertebrates [20]. Human lysozyme was the first mammalian lysozyme to be sequenced and served as a model protein for a wide variety of studies [32]. In the recent years, little evidence on the involvement of lysozymes in malaria pathogenesis has emerged from few studies in *Anopheles* mosquito vector and human host. In *Anopheles gambiae*, *stephensi*, and *dirus*, lysozyme was shown to bind to oocysts of *Plasmodium berghei* and *falciparum* and therefore facilitate their development within the mosquito [23], [33]. In humans, plasma levels of lysozyme, along with those of mieloperoxidase and lipocalin, correlated significantly to degree of parasitaemia, suggesting that the levels of these molecules might be good markers of severe malaria [21], [22]. Moreover, in a recent work published by our group, higher levels of lysozyme were found in supernatants of human monocytes 2 h after phagocytosis of HZ, the malarial pigment produced by *Plasmodium* parasites after haemoglobin catabolism [19]. The present work was conceived and expanded upon this evidence, focusing on mechanisms underlying HZ-dependent induction of lysozyme release.

Preliminar analysis was consistent with our previous results [19], as HZ promoted in a time-dependent manner early lysozyme release (1–2 h after the end of phagocytosis). However, lysozyme release was not increased at later time-points (24–48 h), when a plateau was achieved, possibly as a result of monocyte complete degranulation. Collectively, these time course experiments suggest that HZ induces a very rapid response, and apparently a second wave of lysozyme release is not likely to occur.

Further investigation was aimed at determining what component of HZ could be responsible for early lysozyme induction. HZ has a scaffold structure composed either by the ferric haem or the lipid moiety, which contains large amounts of mostly esterified monohydroxy derivatives (OH-PUFAs: hydoxy-octadecadienoic acids, HODEs; and hydoxy-eicosatetraenoic acids, HETEs), the stable end products of peroxidation of polyenoic fatty acids, generated through non-enzymatic haem-catalysis; the concentrations of OH-PUFA are approximately 90 micromoles per liter RBC equivalents: 13- and 9-HODE and 9-, 12-, and 15-HETE are predominant in HZ, and the estimated concentrations of all HETE isomers are 39 micromoles per liter RBC equivalents [26]. In the present work, lipid-free sHZ and dHZ did not reproduce the effects of HZ on lysozyme early release, suggesting a major role for the lipid moiety of HZ. Nevertheless, it has been recently proposed that the phagocytosis of the packaging digestive vacuole - and not only of HZ released after schizogony - might be at the root of pathway activation in phagocytic cells [34]. Thus, at the moment an *in* *vivo* involvement of the full digestive vacuole as a causative agent of the enhanced lysozyme levels found in plasma of patients with malaria [21], [22] cannot be excluded.

As a following goal, the possible soluble mediators for HZ-dependent lysozyme early release were searched. In our previous study, the involvement of TNFalpha was proposed; however, a likely role for other not yet identified pro-inflammatory molecules was also speculated, as the upregulatory effects of HZ on lysozyme release were only partially mimicked by exogenous recombinant TNFalpha or abrogated by blocking antibodies [19]. Notable, in human phagocytes such as neutrophil granulocytes and monocytes lysozyme is stored, among others, in the so-called gelatinase granules, which also contain MMP-9 [35]. In a series of previous works, we showed that HZ enhances expression, release and activity of human constitutive monocytic MMP-9 [18] but not inducible MMP-2 [36], and that such an enhancement appears to be mediated by several pro-inflammatory molecules, including TNFalpha [37], IL-1beta [38], and MIP-1alpha [39]. The gene expression of these molecules, along with that of other cytokines and chemokines, was previously reported to be upregulated by HZ, and praecox IL-1beta production was suggested to trigger the expression of all other genes [16]. This evidence is strengthened by the data from this work, showing early HZ-enhanced protein release of IL-1beta, TNFalpha and MIP-1alpha; interestingly, HZ-dependent enhancement of IL-1beta and MIP-1alpha production was already significant 1 h after the end of phagocytosis, whereas a significant TNFalpha increase was found only at the latter time-point (120 min).

Moreover, as resulting from experiments with blocking antibodies and recombinant cytokines, IL-1beta and MIP-1alpha appeared to be causally connected to the HZ-dependent induction of lysozyme release, in a manner similar to TNFalpha. Indeed anti-IL-1beta and MIP-1alpha blocking antibodies reduced, although did not fully abrogate, the HZ-dependent induction of lysozyme release; on the other hand, recombinant IL-1beta and MIP-1alpha partially mimicked the effects of HZ, promoting lysozyme release without reaching the HZ-induced levels. Apparently, all three molecules are required as soluble mediators to fulfil HZ-dependent lysozyme upregulation, since the combination of exogenous TNFalpha, IL-1beta and MIP-1alpha induced lysozyme levels similar to those induced by HZ, while mixed anti-TNFalpha, anti-IL-1beta and anti-MIP-1alpha abrogated HZ-induced lysozyme release. These data are consistent with previous documents showing lysozyme gene dependence on cytokine levels [40], [41].

Nevertheless, it should be critical to underline that, contrary to lysozyme, HZ-dependent enhanced production of TNFalpha, IL-1beta and MIP-1alpha appears to be continuous and to go on also at longer times, as demonstrated by previous works from our group: for instance, 25 ng/ml TNFalpha were measured in HZ-fed monocyte supernatants 72 h after phagocytosis [37]; 35 ng/ml IL-1beta 48 h after phagocytosis [38]; and 20 ng/ml MIP-1alpha 24 h after phagocytosis [39]. Meanwhile, it should be taken in account that HZ-fed monocytes produce increased amounts of several chemotactic molecules other than MIP-1alpha (that is MIP-1beta, GROalpha, GRObeta, GROgamma, MCP-1, IL-8, and ENA-78) [16], and show compromised ability to perform repeated phagocytosis [11]. Thus, in order to translate the present findings into acute and chronic human malaria, and to explain the higher plasma levels of lysozyme found in patients with malaria [21], [22] it is reasonable to hypothesize that the lysozyme accumulation might be a consequence of two complementary events: on the one hand, immediately after HZ phagocytosis human monocytes may release higher amounts of lysozyme; on the other hand, after full release of lysozyme, HZ-laden monocytes could go on producing pro-inflammatory molecules, such as TNFalpha, IL-1beta, MIP-1alpha as well as other chemokines, which might in turn recruit other monocytes into the areas of parasite sequester in microvessels. Therefore, as a consequence of increased circulating levels of TNFalpha, IL-1beta, and MIP-1alpha, and as a result of additional phagocytosis of HZ by newly recruited monocytes (but not of repeated phagocytosis by those already laden), new lysozyme release would be induced, thus contributing to enhance total lysozyme circulating levels.

As a next step, the mechanisms of signal transduction underlying HZ-dependent lysozyme enhancement were investigated. To date, the pathways activated by HZ in human monocytes have been scarcely described so far, although some helpful information comes from several murine models. According to few *in vitro* and *in vivo* studies, an involvement of MAPK in malaria appears to be likely. In murine macrophages or monocytes, HZ was shown to induce activation of p38 [42], and ERK1/2 [43]–[45], but not JNK-2/STAT-1 [43], [44] pathways, whereas *Plasmodium falciparum* glycosylphosphatidylinositol (*Pf*GPI) promoted phosphorylation of all routes [46], [47]. Interestingly, inhibition of *Pf*GPI-dependent activation of MAPK decreased inflammatory responses and enhanced phagocytic clearance of IRBC in mice infected by *Plasmodium berghei* or *chabaudi chabaudi* [48]. In human monocytes, the effects of HZ on MAPK regulation have not been described yet; however, Lucchi et al. recently reported in syncytiotrophoblast cells a HZ-dependent phosphorylation of ERK1/2 and an IRBC-dependent phosphorylation of JNK-1; both events were causally related to production of pro-inflammatory molecules (TNFalpha, MIP-1alpha, IL-8) [49], [50]. The present study shows for the first time that HZ activates p38 MAPK pathway in human monocytes, by inducing p38 MAPK phosphorylation without altering basal protein levels. Such an event is directly connected to HZ-induced lysozyme levels, as demonstrated by experiments using a specific synthetic inhibitor of p38 signalling (SB203580), which abrogated the effects of HZ on lysozyme activity in monocyte supernatants. These observations are consistent with previous data correlating activation of MAPK pathways to degranulation from human neutrophils [51] and p38 MAPK phosphorylation to lysozyme release in vibrio-infected mussel granulocytes [52].

In addition, c-lysozyme gene has been reported to be regulated also through NF-kappaB signaling [53]. Interestingly, little evidence on HZ-dependent NF-kappaB activation is presently available: in human monocytes, HZ was shown to upregulate *in vitro* MMP-9 through long-term (24h after phagocytosis) I-kappaBalpha degradation and NF-kappaB nuclear translocation [27], [54], whereas in murine macrophages either HZ or sHZ promoted cytokine/chemokine production through NF-kappaB activation *in vitro* and *in vivo* [43]–[45]. In the present work an early activation of NF-kappaB pathway in human monocytes was found 2 h after phagocytosis of HZ, which induced cytosolic I-kappaBalpha phosphorylation and degradation, p50/p65 NF-kappaB subunits nuclear translocation and NF-kappaB/DNA binding. All these effects of HZ were abrogated by quercetin, artemisinin, and parthenolide, three molecules which have been reported as NF-kappaB inhibitors [29]–[31] and showing antimalarial properties [55], [56]. Moreover, quercetin, artemisinin, and parthenolide inhibited the HZ-dependent enhancement of lysozyme levels in monocyte supernatants, suggesting a potential role for NF-kappaB in lysozyme regulation by HZ. Interestingly, in a previous work these three inhibitors abrogated the upregulating effects of HZ on MMP-9, TNF and IL-1beta production by human monocytes [27].

In conclusion, the present study shows that phagocytosis of HZ promotes an early cytokine/chemokine-mediated lysozyme release from human monocytes through early activation of p38 MAPK and NF-kappaB pathways. Since the lipid moiety of HZ appears to be involved in lysozyme early release, future studies should be aimed at investigating what lipids might be responsible for such an induction. Interestingly, the lipid moiety of HZ was previously shown to induce TNFalpha, IL-1beta and MMP-9, but not MIP-1alpha, and a major role for 15-HETE was speculated [16], [38], [57]. Moreover, the interaction between IL-1 and HETEs was associated with activation of NF-kappaB and MAPK pathways [58]. Thus, a role for 15-HETE could be likely also in HZ-induced lysozyme release. Collectively, the findings of the present work support the hypothesis suggesting a potential role for lysozyme as an early marker of disease severity in *falciparum* malaria, and might be useful in order to design in the future specific diagnostic approaches for severe malaria.
